# A Case Control Study to Evaluate the Impact of Colchicine on Patients Admitted to the Hospital with Moderate to Severe COVID-19 Infection

**DOI:** 10.1155/2020/8865954

**Published:** 2020-10-27

**Authors:** Tegveer Sandhu, Arlene Tieng, Sridhar Chilimuri, Giovanni Franchin

**Affiliations:** Department of Internal Medicine, BronxCare Health System, Bronx, NY 10457, USA

## Abstract

**Background:**

Colchicine has been used in conditions such as periodic febrile illness, acute pericarditis, and gouty arthritis, all having a common hyperinflammatory response as seen in moderate to severe forms of coronavirus disease 2019 (COVID-19). This project was carried out during the rapid surge of cases in New York City, and the goal was to assess the efficacy of colchicine in treating patients with COVID-19.

**Methods:**

Patients admitted to two distinct pulmonary oriented floors of the BronxCare Hospital Center were compared. Patients on one floor were given colchicine in addition to standard of care, while control patients from another floor received only standard of care. Patients who had at least two separate timepoint measurements for at least two out of four serum inflammatory markers (*C*-reactive protein (CRP), *D*-dimer, ferritin, or lactate dehydrogenase (LDH)) were selected for the final comprehensive analysis.

**Results:**

An initial analysis performed on all patients, irrespective of the availability of two timepoint inflammatory markers, revealed a lower mortality (49.1% versus 72.9%, *P* = 0.002), a lower percentage of intubations (52.8% versus 73.6%, *P* = 0.006), and a higher discharge rate (50.9% versus 27.1%, *P* = 0.002), in the patients who received colchicine. Patients in the final comprehensive analysis groups (34 in the colchicine group and 78 in the control group) had a similar prevalence of comorbid medical conditions, except for renal failure, which was higher in the control group (65.3% versus 35.2%, *P* = 0.015). HTN (71.8% versus 52.9%, *P* = 0.053) and DM (51.3% versus 32.4%, *P* = 0.064) were also more prevalent in the control group, although the difference was not statistically significant. Patients who received colchicine had a lower mortality than the control group (47.1% versus 80.8%, *P* = 0.0003), lower rate of intubations (47.1% versus 87.2%, *P* < 0.0001), and a higher discharge rate (52.9% versus 19.2%, *P* = 0.0003). Patients in the colchicine group also showed a more significant decrease in inflammatory markers for *D*-dimer (*P* = 0.037), CRP (*P* = 0.014), and ferritin (*P* = 0.012).

**Conclusions:**

Our study demonstrates that colchicine improved outcomes in patients with COVID-19 receiving standard of care therapy. Future randomized, placebo-controlled clinical trials to assess the potential benefit of colchicine in COVID-19 are warranted.

## 1. Introduction

Coronavirus disease 2019 (COVID-19) is a rapidly spreading pandemic disease with the number of cases in the United States exceeding four and a half million, along with a rising death toll [1]. Mechanisms behind the mortality of patients with COVID-19 are still incompletely understood, which lead to challenges in determining optimal treatment strategies for patients. Inflammatory cytokine storm and a state of hypercoagulability are two main important pathophysiological mechanisms behind the clinical manifestations of COVID-19, with different treatment approaches towards both.

Cytokine storm phenomenon is observed in many infectious and noninfectious diseases. It represents an excessive release of inflammatory cytokines and is thought to be responsible for the acute lung injury seen in COVID-19 [2]. Increased levels of inflammatory cytokines such as tumor necrosis factor-alpha, interleukin-1, interleukin-6, and interleukin-10 are found in patients with severe COVID-19 [3]. In particular, an increased level of interleukin-6 has been shown to be associated with poor outcomes in patients with COVID-19 [4].

Colchicine is an antimitotic drug, which has been used in the treatment of conditions such as periodic febrile illnesses, familial Mediterranean fever, acute pericarditis, and gouty arthropathy, which are characterized by a hyperinflammatory response due to multiple cytokine activation, as seen in COVID-19. It is a well-tolerated medication with a mild side effect profile, mostly gastrointestinal (abdominal pain, nausea, vomiting, and diarrhea) when present. In rare instances, blood dyscrasias such as leukopenia and pancytopenia have been reported [5]. In the early weeks of widespread infection rate in New York City with an overwhelming presentation of severe COVID-19 cases, a lack of proven therapies prompted our development of a quality improvement project, based on the premise that colchicine, an easy to use, benign, and easily available medication, might help improved outcomes of patients with COVID-19.

## 2. Materials and Methods

### 2.1. Study Design and Setting

This project was designed as a prospective comparative cohort study of patients with a confirmed diagnosis of COVID-19 and admitted to two distinct pulmonary oriented floors of the BronxCare Hospital Center in New York City from March 21, 2020 to May 02, 2020. Both floors are staffed by pulmonary and critical care-trained attendings, a pulmonary fellow, and internal medicine residents. As the number of COVID-19-positive patients increased exponentially towards the end of March, both floors were dedicated exclusively to COVID-19-positive patients.

### 2.2. Cases

Patients admitted to the designated colchicine floor between March 21, 2020 and May 02, 2020, who were 18 years old or older, had clinically suspected COVID-19, or a positive SARS-CoV-2 nasal swab PCR, were considered for colchicine use. A verbal informed consent was obtained from the patients prior to the use of colchicine. Exclusion criteria included pregnancy, end-stage renal disease (ESRD), concurrent use of protease inhibitor, ketoconazole, cyclosporine, or clarithromycin. Dosing was 0.6 mg twice a day for three days and then 0.6 mg once a day for a total of 12 days. Colchicine would not be continued if the patients were discharged from the hospital before completing 15 doses. Doses were adjusted for reduced glomerular filtration rate (GFR) due to chronic kidney disease (CKD) and were stopped if the patients developed a new renal failure or if there was a progression of preexisting CKD. For this study, out of all the patients who received colchicine, only patients who were confirmed positive for SARS-CoV-2 nasal swab PCR were included. To avoid possible confounders from another case-control study underway on the use of lamivudine and dolutegravir in COVID-19, patients who received either lamivudine or dolutegravir were excluded from the study. Patients who received concurrent tocilizumab or convalescent plasma were also excluded. Of all the patients who met the above criteria, only the patients who had at least two separate timepoint measurements for at least two out of four inflammatory markers (*D*-dimer, CRP, LDH, or ferritin) were included in the final comprehensive analysis colchicine group.

### 2.3. Controls

The control cohort is represented by patients admitted to the designated control floor between March 21, 2020 and May 02, 2020, who were 18 years old or older and had a confirmed positive SARS-CoV-2 nasal swab PCR. Patients who received colchicine, lamivudine, dolutegravir, tocilizumab, or convalescent plasma were excluded from the control group. Only patients who had at least two separate timepoint measurements available for at least two out of four inflammatory markers (*D*-dimer, CRP, LDH, or ferritin) were included in the comprehensive analysis control group.

### 2.4. Data Collection

A manual chart review was performed to collect data. Data collected included baseline demographics (medical record number, age, gender, body mass index (BMI)), date of admission, and vital signs at the time of presentation to the hospital (pulse rate, respiratory rate, temperature in Fahrenheit, and oxygen saturation). It was recorded that if the patient was on room air, oxygen by a nasal cannula, a nonrebreather mask, or intubated at the time the initial oxygen saturation was obtained. Further data were collected regarding the hospital course including date of intubation and extubation if mechanically ventilated, data on disposition (whether discharged, expired, or still admitted), number of colchicine doses, other medications if received (hydroxychloroquine, steroids (hydrocortisone, dexamethasone, methylprednisolone, or prednisone were converted to a total dose received of prednisone equivalent), insulin, enoxaparin (inclusive of both prophylactic and therapeutic doses), apixaban, rivaroxaban, warfarin, heparin (subcutaneous or intravenous), or oseltamivir. A chart review was performed for medical history, if present of hypertension (HTN), diabetes mellitus (DM), asthma, chronic obstructive pulmonary disease (COPD), hyperthyroidism, hypothyroidism, and coronary artery disease (CAD). Renal failure was defined as an estimated glomerular filtration rate (eGFR) less than 60 ml/min/1.73 m^2^, at the time of presentation to the hospital, based on the initial labs drawn at the time of admission to the hospital. This quality improvement project was initially approved by the COVID-19 protocol development task committee and subsequently reviewed by the hospital's institutional review board.

### 2.5. Inflammatory Markers

A manual chart review was performed to record the first and last available values for the four serum inflammatory markers (*D*-dimer, CRP, LDH, or ferritin, whichever available), that have been shown to be associated with disease severity in COVID-19. Any inflammatory marker with only one timepoint measurement was disregarded. Only the inflammatory markers with at least two timepoint measurements were recorded. A delta value was calculated by subtracting the last value from the first. A delta percentage was calculated by dividing the difference value with the initial value.

### 2.6. Statistical Analysis

Statistical analyses were performed using IBM SPSS Statistic Version 27 (IBM Corp. and others, 1989, 2019). Dichotomous variables were reported as raw values and percentages and were compared using the *χ*^2^ test. In cases in which the 2  ×  2 matrices contained cells with expected values less than 5, the Fisher exact test was used. The Shapiro–Wilk test was used to check for normality of the continuous variables. For normally distributed variables, independent samples *t*-test was used to check for statistical significance. For the continuous variables that were not normally distributed, we used the Mann–Whitney *U* test to check for statistical significance.

## 3. Results

### 3.1. Study Population

**Figure 1:**
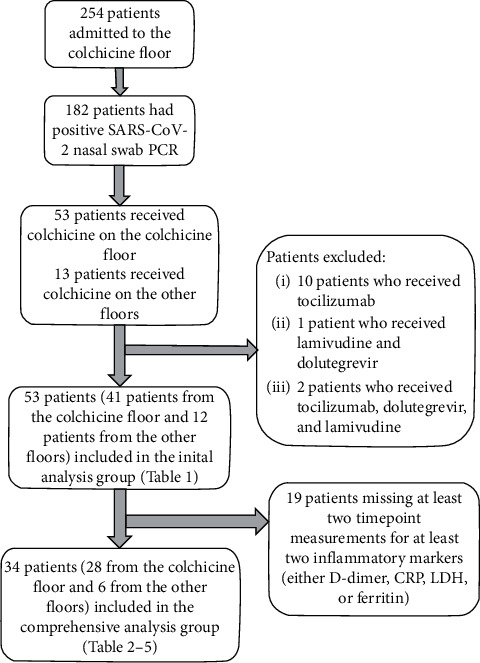
A flow diagram of the selection process for the patients in the colchicine group.

As shown in Figure 1, a total of 254 patients were admitted to the colchicine floor, with 41 patients positive for SARS-CoV-2 nasal swab PCR and meeting the eligibility criteria for the colchicine group (receiving at least one dose of colchicine and not receiving lamivudine, dolutegravir, tocilizumab, or convalescent plasma). In addition, 12 patients from other hospital floors also met the above criteria. Out of a total of 53 patients in the colchicine group, 34 patients had two separate timepoint values for at least two out of four serum inflammatory markers (*D*-dimer, CRP, LDH, or ferritin) and were included in the comprehensive colchicine group analysis.

**Figure 2:**
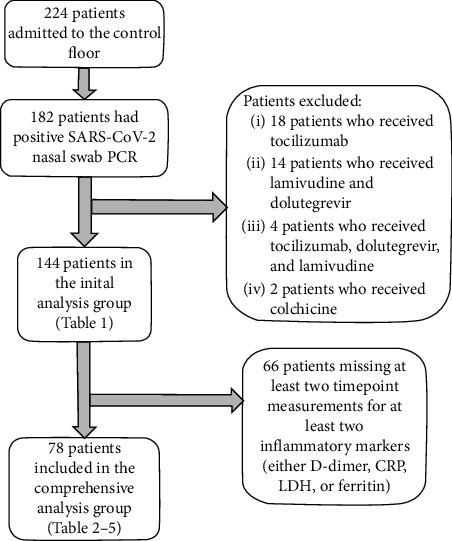
A flow diagram of the selection process for the patients in the control group.

As shown in Figure 2, a total of 245 patients were admitted to the control floor, out of which 144 patients had positive SARS-CoV-2 nasal swab PCR and did not receive colchicine, lamivudine, dolutegravir, tocilizumab, or convalescent plasma. Out of the 144 patients, 78 patients had two separate timepoint values for at least two out of four serum inflammatory markers (*D*-dimer, CRP, LDH, or ferritin) and were included in the comprehensive control group analysis.

### 3.2. Initial Analysis

The initial analysis of all the patients in the colchicine group and the control group (53 and 144 patients in each group, respectively), including patients with or without two timepoint inflammatory markers, is shown in Table 1. While the median age is higher in the colchicine group as compared to the control group (70 years versus 65 years, *P* = 0.049), gender was not statistically different in the two groups, but there was a higher prevalence of men in the colchicine group (64.2% versus 55.6%). The group of patients receiving colchicine had a lower rate of intubation (52.8% versus 73.6%, *P* = 0.006) and lower mortality compared to the control group (49.1% versus 72.9%, *P* = 0.002). The discharge rate was higher in the colchicine group (50.9% versus 27.1%, *P* = 0.002) as compared to the control group.

### 3.3. Comprehensive Analysis

34 patients in the colchicine group and 78 patients in the control group who had at least two timepoint measurements for at least two out of the four serum inflammatory markers (*D*-dimer, CRP, LDH, or ferritin) were included in the comprehensive analysis.

### 3.4. Clinical Characteristics

There was no significant difference between age, gender, or BMI in the two groups. Both groups had a similar pulse rate, respiratory rate, but the colchicine group has a higher temperature at the time of presentation to the hospital (99.95 degrees F versus 98.8 degrees F, *P* = 0.02). Oxygen saturation at the time of presentation was similar in the two groups, although a higher number of patients in the colchicine group were on oxygen by a nasal cannula when the oxygen saturation was recorded (50% versus 29.5%, *P* = 0.037). The number of patients on room air, a nonrebreather mask, or intubated at the time of initial presentation were similar in the two groups. A higher percentage of patients in the control group had HTN (71.8% versus 52.9%, *P* = 0.053) and DM (51.3% versus 32.4%, *P* = 0.064), although it was not statistically significant. Renal failure was more prevalent in the control group compared to the colchicine group (60.3% versus 35.2%, *P* = 0.015). Prevalence of asthma, COPD, hypothyroidism, and CAD was similar in the two groups (Table 2).

### 3.5. Medications Received

A comparison of medications administered to patients in both groups is shown in Table 3. There was no statistically significant difference between both groups on the use of hydroxychloroquine, steroids, insulin, oseltamivir, and enoxaparin. However, there was a trend for a higher amount of steroid use in the control group. Overall, a similar number of patients in both groups received all-inclusive anticoagulation. On further breakdown, a smaller number of patients in the colchicine group received subcutaneous heparin (14.7% versus 39.7%, *P* = 0.009). There was no statistical significance in the two groups on the use of enoxaparin, direct oral anticoagulants (DOACs), warfarin, and intravenous heparin.

### 3.6. Inflammatory Markers

First and last serum measurements for LDH, ferritin, CRP, and *D*-dimer, during the hospitalization, were recorded. The inflammatory marker measurements obtained between the first level and the last level were disregarded. The delta value was obtained by subtracting the last value obtained during the admission by the first value on admission. The raw delta values and the percentage values (percentage change compared to the initial value, calculated by the following formula: initial value subtracted by the final value/initial value) are depicted in Table 4. Although all inflammatory markers showed a trend of lower raw delta levels in the colchicine group, only ferritin showed a statistically significant lower level in the colchicine group (−63 versus 211, *P* = 0.05). Moreover, the lower percentage delta values for patients in the colchicine group were statistically significant for ferritin (−9.6 versus 19.3, *P* = 0.012), CRP (−44.7 versus 16.8, *P* = 0.014), and *D*-dimer (22 versus 147.3, *P* = 0.037).

### 3.7. Primary Outcomes

Primary outcomes are shown in Table 5. Similar to the initial analysis, patients who received colchicine had a lower rate of intubation (47.1% versus 87.2%, *P* < 0.0001), a lower mortality (47.1% versus 80.8%, *P* = 0.0003), and a higher discharge rate (52.9% versus 19.2%, *P* = 0.0003). The mortality in all intubated patients and duration of hospitalization was not statistically different between the two groups.

## 4. Discussion

Many immunomodulatory agents used in rheumatic diseases are being explored for use in COVID-19 as a treatment option or prevention of the exuberant inflammatory response seen in many patients who progress from mild to more severe forms of the disease. Hydroxychloroquine is one such agent that raises intracellular pH and inhibits the activity of lysosomes in antigen presenting cells (APCs), thereby preventing major histocompatibility complex (MHC) class II-mediated antigen presentation to T cells. Hydroxychloroquine showed in vitro activity against Sars-Cov-2 virus, and initial studies showed lower rates of exacerbation, a shortened disease course, and a higher viral clearance in patients with COVID-19 who received hydroxychloroquine, leading to its widespread use in COVID-19 [6, 7]. However, subsequent randomized clinical trials failed to corroborate the initial findings of the improved outcomes with the use of hydroxychloroquine in COVID-19 [8, 9]. Tocilizumab, a monoclonal antibody against interleukin-6, has shown some efficacy in the treatment of severe COVID-19, although the significant cost of this product can be a limiting factor in its widespread use [10–12].

Colchicine is a well-known medication with a mild side effect profile that has been used in other hyperinflammatory states, including periodic febrile illnesses, familial Mediterranean fever, acute pericarditis, and gouty arthropathy. Colchicine binds to unpolymerized tubulin to form tubulin-colchicine complexes, thereby inhibiting their polymerization. As a result, it blocks the cell division during the metaphase of mitosis. In addition, colchicine also inhibits NLRP3 inflammasome, which is a key mechanism of inflammation in gout, and surprisingly vascular diseases. In studies of acute coronary syndrome (ACS), colchicine has been shown to prevent NLPR3 inflammasome-induced caspase-1 activation, leading to decreased levels of interleukin IL-1b, IL-18, and IL-6 and CRP and a reduction in mortality from major cardiovascular events [13–15]. In patients infected with COVID-19, the presence of increased levels of serum inflammatory markers including IL-6, CRP, LDH, ferritin, and *D*-dimers has been associated with an increased risk of progression to more severe forms of the disease. While some positive results have been obtained with the use of antiviral drugs, including remdesivir, some patients still progress to an uncontrolled dysregulated state with alarming levels of inflammatory markers and cytokines, which is believed to contribute to multiorgan dysfunction and subsequent failure. Our hypothesis was that colchicine may offer a cost-effective alternative to slowing the inflammatory response during COVID-19 infection to prevent progression to a hyperinflammatory state (cytokine storm) while not inducing a global immunosuppression and allowing the host to still carry an effective immune response against the virus [16].

Our study showed improved outcomes in the patients who received colchicine compared to the patients who did not. Lower mortality, a lower rate of intubations, and a higher number of discharges were observed in the patients who received colchicine. These results were observed when comparing all patients receiving colchicine against a control cohort (initial analysis—Table 1) and the comprehensive group analysis looking at only patients who had two or more timepoints of inflammatory markers available (Table 5). Although our study lacks the power of a randomized and double-blind design, assigning treatment groups to specific hospital floors decreased some potential biases. However, as noted in the results section, there were still some patients on separate floors who were given colchicine because of treating physician's clinical decisions. Prior data on the possible efficacy of colchicine in patients with COVID-19 are limited, although there are multiple trials currently underway. A randomized controlled trial on patients hospitalized with COVID-19, conducted by Deftereos et al., looked at the fifty-five patients who received colchicine plus standard of care versus fifty patients who only received standard of care, to compare the clinical outcomes between the two groups. Seven (14%) out of the fifty patients in the control group deteriorated, with one needing noninvasive ventilation, five needing intubations (out of which three died shortly), and one patient died of sudden cardiac arrest. The colchicine group, on the other hand, had only one patient (1.8%) in the group of fifty-five who was intubated and expired [17]. It is important to note that any patients during screening who would inevitably require ventilatory support in the next 24 hours, on the bases of clinical assessment at the time of enrollment, were excluded from the study. 184 patients were assessed for eligibility, out of which 56 were excluded, who may have represented a more severe form of COVID-19. In our study, a significant proportion of patients in both the control and colchicine groups was put on oxygen by a nasal cannula (29.5% and 50%, respectively), a nonrebreather mask (24.4% and 50%, respectively), or intubated (5.1% and 2.9%, respectively) at the time of presentation to the hospital, representing significantly more severe form of COVID-19. Oxygen saturation, despite being on the above interventions was 94% in the control group and 95.5% in the colchicine group. Our initial hospital rate of mortality and requirements of mechanical ventilation were also much higher than the Deftereos study, again suggesting that our population represented a much more severe form of the disease. Nevertheless, we found similar positive effects of colchicine, reducing mortality and intubation rate.

A case series reported by Montealegre-Gómez et al., looking at five patients who were receiving colchicine for iatrogenic allogenosis weeks before they tested positive for COVID-19, reported only mild to no symptoms, while they had multiple close contacts who were hospitalized for severe COVID-19 [18]. Another case report by Gandolfini et al. describes a 52 year old female on immunosuppressive therapy for kidney transplant who had moderate to severe respiratory failure due to COVID-19 but sustained remarkable clinical improvement after receiving colchicine and did not require mechanical ventilation. The patient also exhibited a decrease in IL-6 levels after receiving colchicine, which is consistent with the current literature on the effect on colchicine on inflammatory markers [19]. This case also highlights the importance of the timely administration of anti-inflammatory agents in patients with COVID-19. We hypothesize that patients with progressing or established ARDS may have crossed the point where colchicine would no longer be effective in controlling the inflammatory cascade. This may explain the equally high mortality in intubated patients for both colchicine and control groups (93.8% versus 88.2%, respectively) found in our study. Early administration of colchicine may be paramount in the timely prevention of an acute hyperinflammatory state leading to deterioration.

Corticosteroids represent another agent such as colchicine, which has an anti-inflammatory effect, and has been used to control the hyperinflammatory response in COVID-19. More recently, several randomized controlled trials have shown a clear benefit of corticosteroids in patients with moderate to severe COVID-19 infection. In the CODEX randomized controlled trial conducted by Tomazinine et al., the use of intravenous dexamethasone plus standard of care versus the standard of care alone resulted in a significant increase in the number of days alive and ventilator free days over a 28-day period [20]. Another randomized controlled trial called the REMAP-CAP trial randomized 403 patients into three open-label groups: fixed low-dose hydrocortisone, shock-dependent hydrocortisone, and no hydrocortisone. The primary end points included the number of days patients remain alive and free of the organ support (respiratory or cardiovascular). The study showed that both fixed low-dose hydrocortisone (93% probability) and shock-dependent hydrocortisone (80% probability) were superior to no steroids [21]. Our study shows the benefit of colchicine in addition to steroids, since both the groups in our study received steroids but only the colchicine group received colchicine in addition to the steroids. Further studies are needed to explore this added benefit of colchicine in addition to corticosteroids in patients with moderate to severe COVID-19.

Both groups in our study had a similar prevalence of comorbid illness except for renal failure, which was higher in the control group. Although not statistically significant, HTN and DM were also more prevalent in the control group compared to the colchicine group. As HTN and DM are known risk factors associated with increased mortality due to COVID-19, their higher prevalence in the control group may have contributed towards its worse outcomes [22]. Given the small sample size of our study, larger-scale randomized controlled trials are needed to remove the potential confounding of the results of our study by the unequal distribution of comorbid illnesses.

Medication use was similar in the two groups, including the use of all-inclusive anticoagulation. On the further breakdown of the type of anticoagulant given, more patients in the control group received subcutaneous (prophylactic) heparin. This is likely attributable to the increased prevalence of renal failure in this group, which led to an increased use of subcutaneous heparin versus enoxaparin in this group. Concurrently, a higher number of patients in the colchicine group received enoxaparin, although not statistically significant, which again, is likely due to a lower prevalence of renal failure in this group. Renal failure, defined as an eGFR less than 60 ml/min/1.73 m^2^, in the laboratory test performed at the time of admission, may represent a preexisting CKD or a new AKI related to COVID-19 or an AKI superimposed on preexisting CKD. It is likely that the exclusion of the patients with severe AKI or ERSD in the colchicine group contributed towards lesser patients having renal failure in the colchicine group. COVID-19 has been reported to cause AKI, with the proposed pathogenesis of AKI possibly due to a combined direct viral effect through binding of viral proteins to the angiotensin converting enzyme-2 (ACE-2) receptor and a systemic cytokine hyperinflammatory response [23]. It is possible that the anti-inflammatory effect of colchicine can also play a protective role against the development of renal failure in patients with COVID-19.

Inflammatory markers such as *D*-dimer, CRP, LDH, and ferritin have been used to predict the risk of progression to severe COVID-19 infection [24]. For instance, in a study conducted by Zhang et al., a *D*-dimer level higher than four folds the normal can effectively predict in-hospital mortality in patients with COVID-19 [25]. Another study conducted by Li et al. showed a more dynamic relationship between the *D*-dimer levels and clinical course of COVID-19. They divided patients into three groups based on prognosis. They showed that the group of patients with worse prognosis had an uptrending *D*-dimer level, whereas the *D*-dimer level trended down in the group of patients with an improved prognosis. Their study highlights the importance of monitoring the levels of inflammatory markers over the course of disease [26]. The study conducted by Deftereos et al. showed that patients who received colchicine had lower peak *D*-dimer levels compared to those who did not, although it was not statistically significant. They did not report the change in levels of *D*-dimers over time [17]. Our results demonstrate that the patients receiving colchicine had a more significant percentage decrease in *D*-dimer levels compared to the control group. Direct endothelial injury caused by inflammatory cytokines has been shown to cause a state of hypercoagulability, which can lead to an increase in *D*-dimer levels [27]. Colchicine's anti-inflammatory properties may be responsible for the lower rise of the *D*-dimer levels in patients who received colchicine, which could function as an adjunctive treatment to anticoagulants.

Both the colchicine and the control group in our study showed an overall decrease in LDH levels, but only the colchicine group showed an overall decrease in CRP and ferritin levels in addition to the LDH levels. There are limited data on how the trend of the inflammatory markers such as CRP, LDH, or ferritin affects the outcomes in COVID-19. Our study showing a reduced mortality in the colchicine group with more downtrending inflammatory markers versus the control group may point towards the decrease in the levels of the inflammatory markers being an indicator of an improved prognosis.

We recognize our study has important limitations. First, it was not a randomized or blinded trial. Instead, it was initially developed as a quality improvement project aimed at improving outcomes in patients with COVID-19 during a critical time of rapid influx of patients with active infection. A comparison cohort group was selected from a different medical floor, which may decrease some selection bias. The two different floors used for both groups of patients do not differ in terms of selection of clinical severity of admission. There was a lower prevalence of renal failure in the colchicine group, which may have been due to the exclusion criteria for the use of colchicine. HTN and DM were also more prevalent in the control group, which although not statistically significant, may represent a potential higher risk of progression to severe form of COVID-19.

Another limitation of our study is the sample size. In the detailed analysis group, we had 34 patients in the colchicine group and 78 patients in the control group. The lack of statistically significant difference between the groups for the raw delta values for *D*-dimer, LDH, and CRP may be due to the low number of patients, even though there is a difference in trend, and the percentage delta calculations were statistically significant.

## 5. Conclusion

In conclusion, our results show for the first time that colchicine given to patients admitted to the hospital with COVID-19-related symptoms may improve outcome and is associated with lower levels of inflammatory markers and faster normalization of these markers, including *D*-dimer, LDH, CRP, and ferritin. Our study suggests that colchicine might be an important addition to the armamentarium against COVID-19 and highlights the need for subsequent randomized and double-blinded control trials.

## Figures and Tables

**Table 1:** Initial analysis on all patients who received colchicine and the patients meeting criteria for the control group, irrespective of the availability of two timepoint inflammatory markers. Age was nonnormally distributed; the Mann–Whitney *U* test was used to check for statistical significance. Fisher's exact test was used to check for differences in the distribution of male patients in the two groups. The *χ*^2^ test was used to evaluate for statistical significance of the difference in the intubated patients, patients expired, and patients discharged in the two groups.

	Colchicine group (*n* = 53)	Control group (*n* = 144)	*P* value
Age (median)	70	65	0.049
Gender, male (percentage)	34 (64.2)	80 (55.6)	0.142
Intubated (percentage)	28 (52.8)	106 (73.6)	0.006
Discharged (percentage)	27 (50.9)	39 (27.1)	0.002
Expired (percentage)	26 (49.1)	105 (72.9)	0.002

**Table 2:** Baseline characteristics, vital signs at the time of presentation to the hospital, and underlying comorbidities in comprehensive analysis colchicine and control groups (only including patients having at least two different timepoint measurements for at least two inflammatory markers (either *D*-dimer, CRP, LDH, or ferritin)). Age and pulse rate were normally distributed; the independent *t*-test was used to compare the two groups. BMI, temperature, oximetry (SpO_2_), and respiratory rate were nonnormally distributed; the Mann–Whitney *U* test was used to compare the two groups. The *χ*^2^ test was used to compare the gender, patients on room air, oxygen by a nasal cannula, a nonrebreather mask, the prevalence of HTN, DM, and renal failure in the two groups. Fisher's exact test was used to compare the number of patients intubated at the time of presentation, the prevalence of asthma, COPD, hypothyroidism, and CAD in the two groups.

	Colchicine group (*N* = 34)	Control group (*N* = 78)	*P* value
Demographics			
Age (mean ± SD)	67.7 ± 12.3	66.4 ± 13.3	0.626
Gender, male (percentage)	21 (61.8)	40 (51.3)	0.306
BMI (median)	27.4	27.5	0.613
Vital signs at the time of presentation to the hospital			
Pulse rate (mean ± SD)	99.7 ± 19.1	99.5 ± 20.9	0.973
Respiratory rate (median)	20	22	0.631
Temperature (median)	99.95	98.8	0.020
Initial SpO_2_ (median)	95.5	94	0.247
On room air when initial SpO_2_ obtained (percentage)	9 (26.5)	32 (41)	0.141
On oxygen by a nasal cannula when initial SpO_2_ obtained (percentage)	17 (50)	23 (29.5)	0.037
On a nonrebreather mask when initial SpO_2_ obtained (percentage)	7 (20.6)	19 (24.4)	0.664
Intubated when initial SpO_2_ obtained (percentage)	1 (2.9)	4 (5.1)	1.00
Comorbid illnesses			
HTN	18 (52.9)	56 (71.8)	0.053
DM	11 (32.4)	40 (51.3)	0.064
Asthma	1 (2.7)	8 (10)	0.272
COPD	5 (14.7)	6 (7.7)	0.304
Hypothyroidism	3 (8.8)	3 (3.8)	0.365
CAD	2 (5.9)	6 (7.7)	1.00
Renal failure	12 (35.2)	47 (60.3)	0.015

HTN, hypertension; DM, diabetes mellitus; COPD, chronic obstructive pulmonary disease; CAD, coronary artery disease; CKD, chronic kidney disease.

**Table 3:** Comparison of medications received during the hospitalization across the comprehensive analysis colchicine and control groups. Hydroxychloroquine doses received and total equivalent prednisone dose received were nonnormally distributed. The Mann–Whitney *U* test was used to compare the two groups. The *χ*^2^ test was used to check for differences in the number of patients receiving steroids, insulin, anticoagulation, enoxaparin, subcutaneous heparin, and direct acting oral anticoagulants (DOACs). Fisher's exact test to check for differences in the number of patients receiving hydroxychloroquine, oseltamivir, intravenous heparin, and warfarin.

	Colchicine group (*n* = 34)	Control group (*n* = 78)	*P* value
Hydroxychloroquine	32 (94.1)	68 (87.2)	0.651
Hydroxychloroquine doses received (median)	5	5	0.458
Received steroids	19 (55.9)	47 (60.3)	0.665
Total equivalent prednisone dose received (median)	101.3	177.4	0.467
Insulin	22 (64.7)	62 (79.5)	0.097
Oseltamivir	30 (88.2)	68 (87.2)	1
Received anticoagulation	32 (94.1)	74 (94.9)	0.871
Enoxaparin	28 (82.4)	57 (73.1)	0.291
DOACs	14 (41.2)	19 (24.4)	0.073
Intravenous heparin	1 (2.9)	10 (12.8)	0.168
Subcutaneous heparin	5 (14.7)	31 (39.7)	0.009
Warfarin	0	1 (1.3)	1

**Table 4:** Comparison of median values of inflammatory markers delta and delta percentage between the colchicine and control groups. Delta values were calculated by subtracting the last measurement from the first measurement. Inflammatory marker delta and delta percentage was nonnormally distributed; the Mann–Whitney *U* test was used to check for differences in the two groups.

	Colchicine group (*N* = 34)	Control group (*N* = 78)	*P* value
D-dimer delta	125	721	0.150
D-dimer delta (percentage)	22	147.3	0.037
LDH delta	−118	−61	0.779
LDH delta (percentage)	−21.6	−9.4	0.417
CRP delta	−33	15	0.116
CRP delta (percentage)	−44.7	16.8	0.014
Ferritin delta	−63	211	0.050
Ferritin delta (percentage)	−9.6	19.3	0.012

**Table 5:** Survival or need for mechanical ventilation in comprehensive analysis colchicine and control groups. Hospitalization days were nonnormally distributed. The Mann–Whitney *U* test was used to check for differences in the two groups. The *χ*^2^ test was used to check for differences in the number of intubations, patients expired, and patients intubated in the two groups and mortality in intubated patients.

	Colchicine group (*N* = 34)	Control group (*N* = 78)	*P* value
Hospitalization days (median)	10.5	11	0.947
Discharged (percentage)	18 (52.9)	15 (19.2)	0.0003
Expired (percentage)	16 (47.1)	63 (80.8)	0.0003
Intubated (percentage)	16 (47.1)	68 (87.2)	<0.0001
Mortality in intubated patients (percentage)	15/16 (93.8)	60/68 (88.2)	0.455

## Data Availability

The data used to support the findings of this study are available from the corresponding author upon request.

## References

[1] Centers for Disease Control and Prevention (2020). *Coronavirus Disease 2019 (COVID-19) in the U.S.*.

[2] Zhang W., Zhao Y., Zhang F. (2020). The use of anti-inflammatory drugs in the treatment of people with severe coronavirus disease 2019 (COVID-19): the perspectives of clinical immunologists from China. *Clinical Immunology*.

[3] Diao B., Wang C., Tan Y. (2020). Reduction and functional exhaustion of T cells in patients with coronavirus disease 2019 (COVID-19). *Frontiers in Immunology*.

[4] Shi Y., Wang Y., Shao C. (2020). COVID-19 infection: the perspectives on immune responses. *Cell Death & Differentiation*.

[5] Leung Y. Y., Yao Hui L. L., Kraus V. B. (2015). Colchicine-update on mechanisms of action and therapeutic uses. *Seminars in Arthritis and Rheumatism*.

[6] Wang M., Cao R., Zhang L. (2020). Remdesivir and chloroquine effectively inhibit the recently emerged novel coronavirus (2019-nCoV) in vitro. *Cell Research*.

[7] Gao J., Tian Z., Yang X. (2020). Breakthrough: chloroquine phosphate has shown apparent efficacy in treatment of COVID-19 associated pneumonia in clinical studies. *Bioscience Trends*.

[8] Boulware D. R., Pullen M. F., Bangdiwala A. S. (2020). A randomized trial of hydroxychloroquine as postexposure prophylaxis for Covid-19. *New England Journal of Medicine*.

[9] Shukla A. M., Archibald L. K., Wagle Shukla A., Mehta H. J., Cherabuddi K. (2020). Chloroquine and hydroxychloroquine in the context of COVID-19. *Drugs in Context*.

[10] Luo P., Liu Y., Qiu L., Liu X., Liu D., Li J. (2020). Tocilizumab treatment in COVID‐19: a single center experience. *Journal of Medical Virology*.

[11] Kewan T., Covut F., Al-Jaghbeer M. J., Rose L., Gopalakrishna K. V., Akbik B. (2020). Tocilizumab for treatment of patients with severe COVID-19: a retrospective cohort study. *EClinicalMedicine*.

[12] Guaraldi G., Meschiari M., Cozzi-Lepri A. (2020). Tocilizumab in patients with severe COVID-19: a retrospective cohort study. *The Lancet Rheumatology*.

[13] Martínez G., Robertson S., Barraclough J. (2015). Colchicine acutely suppresses local cardiac production of inflammatory cytokines in patients with an acute coronary syndrome. *Journal of the American Heart Association*.

[14] Deftereos S. G., Siasos G., Giannopoulos G. (2020). The Greek study in the effects of colchicine in COvid-19 complications prevention (GRECCO-19 study): rationale and study design. *Hellenic Journal of Cardiology*.

[15] Tardif J.-C., Kouz S., Waters D. D. (2019). Efficacy and safety of low-dose colchicine after myocardial infarction. *New England Journal of Medicine*.

[16] Beigel J. H., Tomashek K. M., Dodd L. E. (2020). Remdesivir for the treatment of Covid-19—final report. *New England Journal of Medicine*.

[17] Deftereos S., Giannopoulos G., Vrachatis D. (2020). Effect of colchicine vs standard care on cardiac and inflammatory biomarkers and clinical outcomes in patients hospitalized with coronavirus disease 2019. *JAMA Network Open*.

[18] Montealegre-Gómez G., Garavito E., Gómez-López A., Rojas-Villarraga A., Parra-Medina R. (2020). Colchicine: a potential therapeutic tool against COVID-19. Experience of 5 patients. *Reumatología Clínica*.

[19] Gandolfini I., Delsante M., Fiaccadori E. (2020). COVID-19 in kidney transplant recipients. *American Journal of Transplantation*.

[20] Tomazini B., Maia I., Cavalcanti A. (2020). Effect of dexamethasone on days alive and ventilator-free in patients with moderate or severe acute respiratory distress syndrome and COVID-19: The CoDEX randomized clinical trial. *JAMA*.

[21] Angus D., Derde L., Al-Beidh F. (2020). Effect of hydrocortisone on mortality and organ support in patients with severe COVID-19. *JAMA*.

[22] Guan W.-j., Ni Z.-y., Hu Y. (2020). Clinical characteristics of coronavirus disease 2019 in China. *New England Journal of Medicine*.

[23] Adapa S., Chenna A., Balla M. (2020). COVID-19 pandemic causing acute kidney injury and impact on patients with chronic kidney disease and renal transplantation. *Journal of Clinical Medicine Research*.

[24] Chilimuri S., Sun H., Alemam A. (2020). Predictors of mortality in adult population admitted with COVID-19: a retrospective cohort study from New York city. *Western Journal of Emergency Medicine: Integrating Emergency Care with Population Health*.

[25] Zhang L., Yan X., Fan Q. (2020). D‐dimer levels on admission to predict in‐hospital mortality in patients with Covid‐19. *Journal of Thrombosis and Haemostasis*.

[26] Li Y., Zhao K., Wei H. (2020). Dynamic relationship between D‐dimer and COVID‐19 severity. *British Journal of Haematology*.

[27] Notley C., Tinlin S., Sawyer L., Begbie M., Lillicrap D. (2000). The factor VIII acute phase response requires the participation of NF*κ*B and C/EBP. *Thrombosis and Haemostasis*.

